# Investigation of Performance Parameters of Spherical Gold Nanoparticles in Localized Surface Plasmon Resonance Biosensing

**DOI:** 10.3390/mi14091717

**Published:** 2023-08-31

**Authors:** Vivek Semwal, Oliver Rishøj Jensen, Ole Bang, Jakob Janting

**Affiliations:** 1 DTU Electro, Department of Electrical and Photonics Engineering, Technical University of Denmark, DK-2800 Kongens Lyngby, Denmark; 2 Novo Nordisk Park, DK-2760 Måløv, Denmark

**Keywords:** gold nanoparticles, plasmonics, biosensor, aptamer, localized surface plasmon resonance

## Abstract

In this paper, we present numerical and experimental results on Localized Surface Plasmon Resonance (LSPR) refractive index (RI) sensitivity, Figure of Merit (FoM), and penetration depth (*d_p_*) dependence on spherical gold nanoparticles (AuNPs) size, and the effects of AuNP dimer interparticle distance (*d_s_*) studied numerically. These parameters were calculated and observed for *d* = 20, 40, 60, 80, and 100 nm diameter spherical AuNPs. Our investigation shows *d* = 60 nm AuNPs give the best FoM. The AuNP dimer interparticle distance can significantly influence the RI sensitivity. Therefore, the effect of distances between pairs of *d* = 20 nm and 60 nm AuNPs is shown. We discuss the importance of penetration depth information for AuNPs functionalized with aptamers for biosensing in the context of aptamer size.

## 1. Introduction

Nanoparticles transformed the field of sensing, with especially the plasmonic nanoparticles showing their ultrasensitive nature for sensing applications [1,2,3,4]. The combination of plasmonic nanoparticles and biomolecules, such as enzymes, antibodies, DNA, or aptamers are extensively used for biosensing applications [2,4,5,6]. Plasmonic nanoparticles exhibit unique features when interacting with light and are specifically useful for the sensing of ultra-low concentrations of analytes. Gold is one of the materials preferred by researchers for sensing applications due to its unique optical features, easy synthesis, chemical stability, photostability, biocompatibility, and easy surface functionalization [2,6]. Plasmonic nanoparticles can absorb the light in the visible region, and the peak absorbance wavelength strongly depends on the shape and size of the nanoparticles and the surrounding medium’s RI. In the past few decades, for biosensing applications, enzymes and antibodies were the main recognition elements. The enzymes and antibodies commonly used for chemical recognition/sensing are very sensitive to temperature, unstable, and costly, and, therefore, long-term use and storage with temperature fluctuations can affect the performance of these biomolecules, which ultimately affects the performance of the sensor [7,8]. Therefore, at present, aptamers are a good alternative to these biomolecules as they have several attractive properties like superior affinity and specificity to the target, longer shelf life, small size, better stability, they are easy to chemically modify, etc. [7,8]. Aptamers are developed by the SELEX process, which is the systematic evolution of ligands by exponential enrichment invented by Tuerk and Gold in 1990 [7,9]. Aptamers are usually synthesized by selecting them from a large random sequence pool.

The combination of biorecognition molecules like aptamers, antibodies, enzymes, etc. and gold nanoparticles (AuNPs) can be used for sensing applications using the Localized Surface Plasmon Resonance (LSPR) phenomenon. When light impinges on metal nanoparticles, for a particular frequency of the EM wave, the incident light gets coupled to the oscillations of electrons inside the metal nanoparticles and, collectively, the electrons starts to oscillate locally (plasmon) in the nanoparticles. This phenomenon is called LSPR [3,4]. This phenomenon is highly sensitive to the RI of the surrounding medium of nanoparticles [1,3,4]. LSPR offers real-time and label-free sensing at very low RI-changing concentrations of chemicals in the surrounding medium with limited temperature cross-sensitivity [3].

The AuNP based biosensors exist mainly as two types: one, in which the metal nanoparticles are in colloidal form, and a second, where the metal nanoparticles are attached to the substrate, such as an optical fiber, a waveguide, a glass slide, or a chip. Colloidal AuNP sensors are designed such that the binding of an analyte causes particle aggregation to produce color change because aggregate formation results from the electronic dipole–dipole coupling between neighboring particles [10]. This type of sensing is known as colorimetric sensing [11]. In colorimetric sensing, the interaction of the analytes and biomolecules can be recognized with the naked eye. AuNPs are the ideal material for colorimetric sensors because the high extinction coefficient leads to the generation of intense colors. The interesting thing is that this color change can also appear for low concentrations, like in the nM to pM [11] ranges. The second type is LSPR based sensors; the nanoparticles are coated onto a substrate and then the aptamers/biomolecules are immobilized on the metal nanoparticles.

AuNPs have been developed in various shapes, such as spherical, rods, stars, cubes, etc. In this paper, we focused on the spherical AuNPs because these are the most frequently used by researchers. LSPR sensing systems have been developed for the detection of environmental chemicals, toxins, and various biological agents, etc. [1,3]. In colorimetric sensors, the color change depends on the degree of agglomeration. Therefore, the size of the nanoparticle, penetration depth, and FWHM do not affect the performance of the sensor. However, in substrate based LSPR, the sensor’s performance strongly depends on several parameters, such as the particle size, shape, interparticle distance and penetration depth into the surrounding medium. The fabrication of fiber/substrate-based LSPR biosensors requires that these parameters are optimized for better performance. The size of AuNPs affects the sensitivity, penetration depth, FoM, and detection accuracy of the sensor and, therefore, the selection of suitably sized nanoparticles is important. For effective sensing, the aptamer/biomolecules and analytes to be detected should be in the LSPR field penetration depth region of the nanoparticle. The RI sensitivity of the LSPR sensor is defined as the change in the peak absorbance wavelength divided by the change in the RI of the surrounding medium of nanoparticles. We know that sensitivity increases with particle size, but the broadening also increases; there is a tradeoff between the sensitivity and broadening. Therefore, the FoM would be the more correct way to characterize the performance of the sensor. FoM is defined as the ratio of the sensitivity of the sensor to the FWHM of the absorbance curve. Thus, a broad absorbance curve decreases the detection accuracy of the sensor. In this paper, we present a study of the effect of the size of the spherical AuNPs on the sensitivity, the FoM, and the penetration depth around the nanoparticle. The electric field penetration depth for the different sizes of nanoparticles is crucial for the selection of nanoparticles in relation to the different-sized analytes/aptamers. The interparticle distance between the nanoparticles strongly influences the electric field and affects the performance of the sensors. The combination of two nanoparticles close to each other is called a dimer, and the electric field enhancements in the nanogap between the particles are known as hot spots. It is observed that the hot spots can be enhanced by decreasing the inter-particle distance until reaching the quantum tunneling region [12]. Therefore, we have also investigated the effect of interparticle distance (*d_s_*) for *d* = 20 and 60 nm AuNP dimers. In recent years, several research articles have reported on the performance parameters of AuNPs because of their interesting biosensing applications [13,14,15,16]. Chen et al. [13] presented the shape- and size-dependent refractive index sensitivity of AuNPs. The refractive index sensitivity of the AuNP dimers has been explored [15,16]. These reported papers mainly focused on a particular parameter, such as size- and shape-dependent RI sensitivity, RI sensitivity of dimers, and RI sensitivity of dimers limited to a particular size. In most studies, the important biosensing performance parameters of AuNPs have not been presented. Particularly, the penetration depth information is not discussed in most of the reported papers [13,14,15,16].

To the best of our knowledge, there is a lack of systematic studies on the spherical AuNP’s performance parameters and its dependence on size. In the literature, the particle diameter range *d* = 20 to 100 nm seems to be of highest interest for biosensing. Therefore, we have investigated the performance parameters, such as sensitivity, FoM, penetration depth, and effect of interparticle distance in dimers of spherical AuNPs in that size range. The goal of this study is to fill this gap by providing a quantitative analysis of the plasmonic sensing properties of AuNPs, which can be useful for researchers to design efficient LSPR sensors.

## 2. Materials and Methods

The AuNPs were purchased from Sigma Aldrich (St. Louis, MO, USA) in 25 mL bottles in 0.1 mM PBS. The studied particle diameters were 20 nm (product no 753610), 40 nm (product no 753637), 60 nm (product no 753653), 80 nm (product no 753661), and 100 nm (product no 753688). Methanol (34885-1L), Acetone (product number 179124), Isopropyl alcohol (IPA) (product number W292907), and Acetonitrile (product number 271004) were also purchased from Sigma Aldrich. Quartz cuvettes (product name CV10Q700F) for UV-VIS absorbance measurements were purchased from Thorlabs. The experimental UV-VIS data were collected using an Ocean Insight (Geograaf, EW Duiven, The Netherlands) HR2000+ spectrometer (product name HR2000CG, Ocean Insight). An Ocean Insight CUV-ALL-UV cuvette holder (product name CUV-UV, Ocean Insight) was used. The light source used was a DH-2000 BAL deuterium halogen light source (product number DH-2000-BAL, Ocean Insight).

## 3. Numerical Modelling Analysis

**Figure 1:**
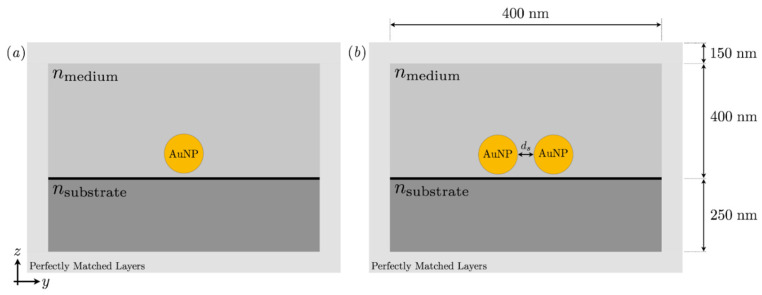
(**a**) A sketch of the system, which was simulated in COMSOL. A single AuNP was placed 1 nm above a PMMA substrate. (**b**) Two AuNPs were placed 1 nm above a PMMA substrate and separated by a distance *d_s_*. The AuNPs were surrounded by some medium with RI = *n_medium_*.

To evaluate the optical properties of the AuNPs, numerical modeling was performed in COMSOL Multiphysics 5.6 [17]. The hardware used was an Intel Core i5-10500 3.10 GHz CPU, and the installed memory capacity (RAM) was 16 GB. The physical 3D geometry is modeled by a box containing a substrate, spherical AuNPs, and a surrounding medium. For numerical reasons, a Perfectly Matched Layers (PML) is added to mimic an open and nonreflecting infinite domain. Optical properties of PMMA were assigned to the substrate to mimic an optically transparent material. Two systems were modeled, one with a single AuNP, shown in Figure 1a, and one where two AuNPs were separated by a distance ds shown in Figure 1b.

For the medium and substrate domains, we use only the real part of the RI. The RI of the substrate was set to 1.492 (PMMA). For the attachment of the AuNPs on a PMMA substrate, generally, an (3-Aminopropyl)triethoxysilane (APTES) layer is used for the binding of AuNPs to the surface. The thickness of the APTES layer is on the order of a few Å [18], typically in the range 0.6–1.9 nm in aqueous media [19]. In this work, the height from the substrate to the AuNP was set to 1 nm. The silane layer was not considered in the model because, in the liquid, it will not be completely uniform. The complex relative permittivity function for gold was obtained from a paper by A. Attaran et al. [20]. The data were analyzed in ImageJ 1.53 m and interpolated in COMSOL. The relative permeability is set to one, and the electrical conductivity is set to zero for all domains. The temperature was set to room temperature, 293.15 K. The COMSOL module used was electromagnetic waves, frequency domain. The incident electric field for excitation of the AuNPs was a y-polarized plane wave propagating in the z-direction with an angle of incidence of zero to the substrate. The input port was set to the boundary between the PMMA substrate and PML. Further, the output port was set to the boundary between the medium and PML. A Floquet periodicity was used as a periodic boundary condition. From the scattered field formulation, all cross-section quantities of interest, such as absorption, scattering, and extinction, can be computed, and the associated electric field norm can be extracted. A mesh convergence test was conducted, and it was found that the optimal mesh structure was for a maximum element size of 40 nm and minimum element size of 4 nm. The mesh for the PML was set to a fixed number of elements of 5.

## 4. Experimental Setup

**Figure 2:**
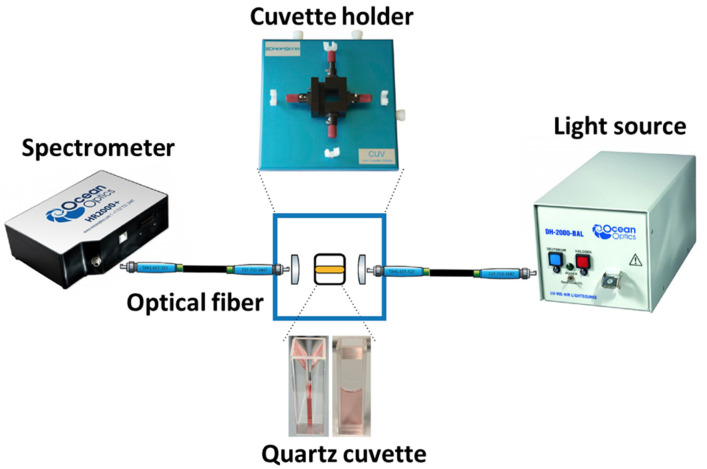
Schematic diagram of experimental setup for UV-VIS study of AuNPs.

The experimental setup is shown in Figure 2. It consists of an ocean optics CUV-UV cuvette holder with a 1 cm pathlength quartz cuvette. For measurement of the absorbance spectra, a tungsten halogen lamp and a spectrometer are connected to the CUV-UV cuvette holder via optical fiber cables with SMA connectors.

## 5. Results and Discussion

### 5.1. RI Sensitivity, Figures of Merit of Different Size AuNPs

The RI sensitivities of different sized nanoparticles (*d* = 20, 40, 60, 80, and 100 nm) were investigated by simulations and experiments. First, we calculated the RI sensitivities of the different-sized AuNPs via simulations, and then we performed the experiments. For the RI variation around the AuNPs, we changed the surrounding medium of the AuNPs by placing the AuNPs in different solvents of different RIs, namely PBS (1.339), Acetonitrile (1.3441), Acetone (1.358), and isopropanol (1.3776). We performed this by centrifuging 400 µL PBS containing AuNPs, removing 380 µL of the PBS from the solution, and then adding the same amount (380 µL) of different solvents like acetonitrile, acetone, and IPA to it. The pictures of AuNPs (*d* = 20, 40, 60, 80, and 100 nm) in different solvents are shown in Figure S1 in the Supplementary File.

**Figure 3:**
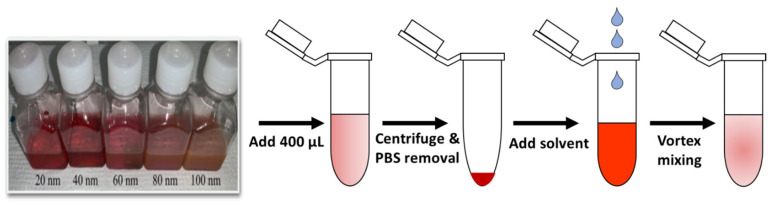
Preparation steps for AuNPs in different solvents.

**Figure 4:**
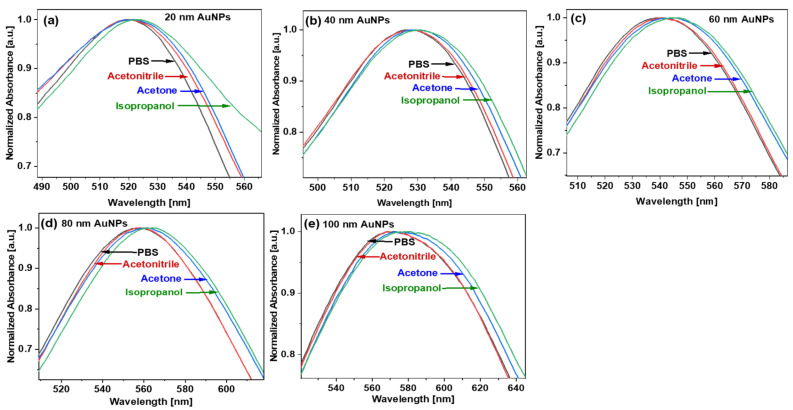
Experimental UV-VIS spectra of AuNPs in different solvents with nanoparticles with average diameters of (**a**) 20 nm, (**b**) 40 nm, (**c**) 60 nm, (**d**) 80 nm, and (**e**) 100 nm.

Figure 3 shows a schematic diagram of the preparation steps for AuNPs in different solvents. The absorbance curves of *d* = 20, 40, 60, 80, and 100 nm AuNPs at different RI mediums are shown in Figure 4. We can see that, as the RI around the nanoparticles increases, the peak absorbance wavelength shifts towards the higher wavelength side. The absorbance curves of *d* = 20, 40, 60, 80, and 100 nm AuNPs from 400 nm to 750 nm at different RI mediums are shown in Figure S2 in the Supplementary File.

**Figure 5:**
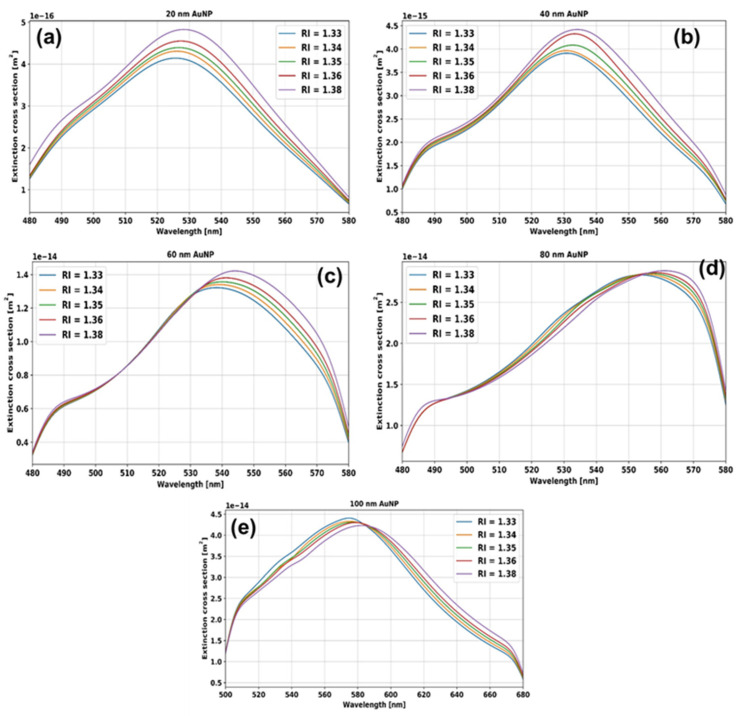
Simulated extinction spectra of AuNPs in different RIs with nanoparticle diameters of (**a**) 20 nm, (**b**) 40 nm, (**c**) 60 nm, (**d**) 80 nm, and (**e**) 100 nm.

**Figure 6:**
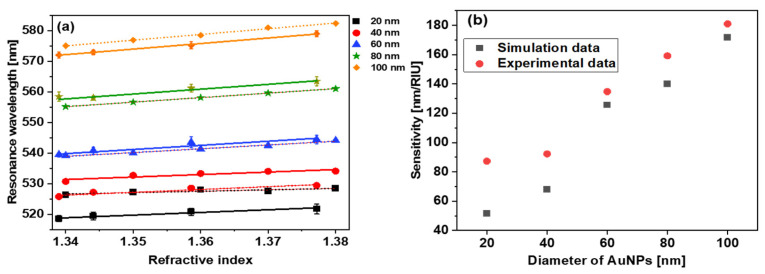
(**a**) Numerically (dotted line) and experimentally (solid line) found resonance wavelength vs. RI. (**b**) numerically (black squares) and experimentally (red dots) found sensitivity vs. diameter of AuNPs.

The extinction curves of *d* = 20, 40, 60, 80, and 100 nm AuNPs at different RI mediums are shown in Figure 5. The resonance wavelengths of AuNPs in different solvents were extracted from Figure 4 and Figure 5, and the data points are plotted in Figure 6. Figure 6a shows the simulated (dotted line) and experimental (solid line) relation between the RI of the surrounding medium and the resonance wavelength for *d* = 20, 40, 60, 80, and 100 nm AuNPs. By fitting these data points, the RI sensitivity has been calculated for different sized nanoparticles by calculating the slope of the fitting data points. The graph between resonance wavelength and RI looks linear in Figure 6a. The error bars in the experimental points were calculated by taking the standard deviation of the resonance wavelengths obtained after repeating the measurements three times. It is notable that the sensitivity of the nanoparticles is size-dependent and increases with the size of the AuNPs. The numerically and experimentally found sensitivities of *d* = 20, 40, 60, 80, and 100 nm AuNPs are shown in Figure 6b. For small-size AuNPs, the simulated and experimental results deviate the most because these particle sizes are less uniform. For *d* = 60, 80, and 100 nm sized nanoparticles, the experimental and simulated values are in better agreement. The absorbance curve of AuNPs starts from 55 to 60% of the maximum absorbance value. Therefore, we have not calculated the FWHM but the full width of the absorbance curve at 60, 70, and 80% value of maximum absorbance. We have calculated the full width of the LSPR absorbance of *d* = 20, 40, 60, 80, and 100 nm AuNPs at these absorbance levels. The trend of the absorbance curve width for *d* = 20, 40, 60, 80, and 100 nm AuNPs in the experiments and simulations are similar. The absorbance curve width increases with the size of the AuNPs, which increases the uncertainty of the peak absorbance wavelength and, hence, decreases the RI sensing accuracy.

**Figure 7:**
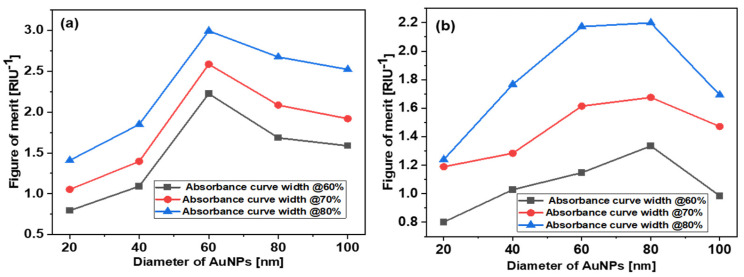
Dependence of FoM on AuNP diameter. (**a**) Simulations, (**b**) experiments.

Figure 7a shows the simulated values of the FoM for the *d* = 20, 40, 60, 80, and 100 nm AuNPs, while Figure 7b shows the experimental values for the same. As can be seen, the experimental and theoretical FoM data points for different sized nanoparticles also present similar trends. Initially, the FoM increases with the size of AuNPs and then decreases. From simulation, the best FoM was observed for the 60 nm AuNPs. The experimental FoMs are also in good agreement with the simulated results, and the *d* = 60 and 80 nm AuNPs show the best FoM. These results indicate that, for spherical AuNPs, around *d* = 60–80 nm AuNPs give a better FoM. For different shapes, such as rods, stars, cubes, and prisms, it could be different.

The simulated and experimental values of the FoM are slightly different. The reason for this difference is the size distribution of the AuNPs used in the experiments. As per the supplier (Sigma Aldrich), the variation in the size of the AuNPs is <12%, whereas, in the simulation, we considered a fixed dimensions.

### 5.2. Localized Plasmon Penetration Depth for Spherical AuNPs

**Figure 8:**
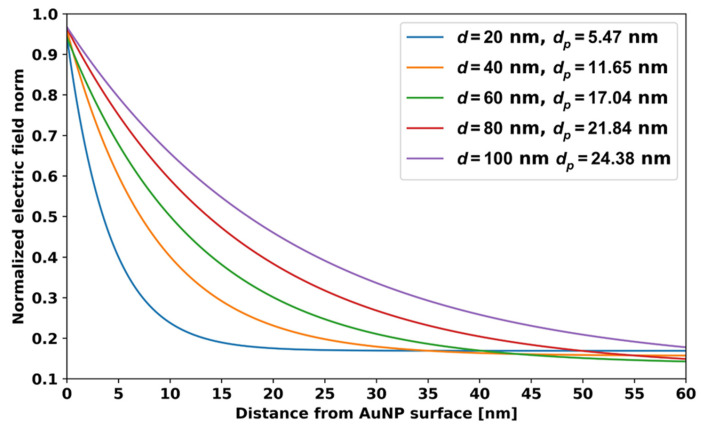
LSPR electric field and penetration depth *d_p_* for AuNPs with different d.

The penetration depth is a crucial parameter for the AuNP-based LSPR sensors. LSPR/SPR sensors can only detect surrounding RI changes when they occur near the electric field around the nanoparticles or the thin film. The LSPR electric field decays exponentially from the surface of the nanoparticles, and, therefore, it can reach up to only very short distances. The electric field strength around the nanoparticles at 1/*e* to its maximum value is called the penetration depth, *d_p_*. Theoretically, we have investigated the electric field penetration depth for the different sized AuNPs. This penetration depth information is useful in choosing the optimal AuNP size for specific aptamer and analyte molecular sizes. Figure 8 shows the normalized electric field vs. the distance from the *d* = 20, 40, 60, 80, and 100 nm AuNPs. From this, we can see the electric field is maximum at the surface of the AuNPs and decreases exponentially as we move out from the surface of the AuNPs. We fitted the data to an exponential function of the form *f*(*x*) = *Ae*^−*Bx*^ + *C*, where *x* is distance, and *A*, *B*, *C* are constants, and we then calculated the distance *d_p_* at which *f*(*x*) is equal to *f*(0)/*e*. The penetration depths, *d_p_,* for 20, 40, 60, 80, and 100 nm diameter nanoparticles are 5.47, 11.65, 17.04, 21.84, and 24.38 nm, respectively.

Aptamers are usually 20- to 60-nucleotide-long chains. The length of one nucleotide unit is around 3.3 Å (0.33 nm) [21]. Using this unit length of nucleotides, one can easily estimate the length of the aptamer. When an aptamer captures an analyte molecule, it is folded, and its length reduces [22]. It is recommended that the size of the folded aptamer should always lie reasonably within the electric field around the nanoparticles. The length of a sequence of 40 nucleotides is around 12 nm. When the aptamer captures a molecule, its length reduces around 40–50% [22], and then the 40-nucleotide-long aptamer will extend to a distance of up to 6 nm from the surface of the AuNPs, which is close to the *d_p_* = 5.47 nm for the *d* = 20 nm AuNPs. If the analyte molecules have a size of 100 nm, then *d* = 20 to 100 nm spherical AuNPs cannot be recommended for sensing because the penetration depth of these AuNPs is small compared to the size of the analyte. Therefore, in this situation, instead of spherical AuNPs, other shapes of AuNPs or SPR should be utilized for sensing. From this discussion, it is clear that the penetration depth information is very useful for selecting the appropriate size of the nanoparticles for the desired target molecule and aptamer.

### 5.3. Effect of Interparticle Distance

**Figure 9:**
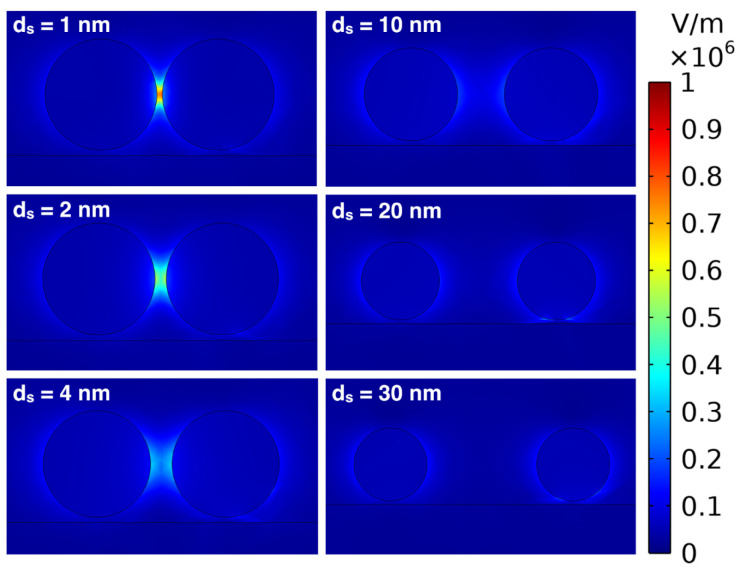
Images of the simulated distributions of electric field norm plotted for 1, 2, 4, 10, 20, and 30 nm distances (*d_s_*) between the *d* = 20 nm AuNPs.

When two nanoparticles are placed close to each other, their LSPR induced electric fields start to interact with each other. The interaction leads to an enhanced electric field between the AuNPs, and the RI sensitivity increases drastically. Figure 9 shows the electric field around dimers of *d* = 20 nm AuNPs with different distances (*d_s_*) between the particles. Here, we can notice that when the distance between the *d* = 20 nm AuNPs is *d_s_* = 20 and 30 nm, the electric fields do not significantly interact, but, when the distance between the *d* = 20 nm AuNPs decreases to around 10 nm, the electric fields start to interact, and the combined electric field strength increases significantly. For small distances of around *d_s_* = 1 to 5 nm, the combined electric field is very high.

**Figure 10:**
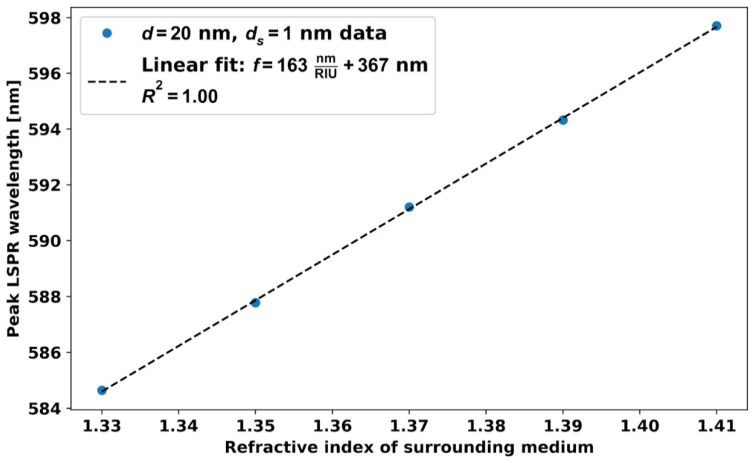
Variation of the resonance wavelength with RI around the *d* = 20 nm AuNP dimer for *d_s_* = 1 nm.

Figure 10 shows the variation of the resonance wavelength with RI around a AuNP dimer composed of *d* = 20 nm AuNPs separated by *d_s_* = 1 nm. The RI sensitivity of the *d* = 20 nm AuNPs dimer with *ds* = 1 nm is 163 nm/RIU, which is close to a 3-fold enhancement from the sensitivity obtained with a single or many well isolated *d* = 20 nm AuNPs. We performed these simulations only down to *d_s_* = 1 nm between the AuNP dimer because, for less than 1 nm distance, quantum tunneling is possible [12]. From the simulation, we have found that *d* = 60 nm diameter AuNPs give the best FoM among the considered 20–100 nm spherical AuNPs, and, therefore, we have simulated the effect of *d* = 60 nm AuNPs interparticle distance.

**Figure 11:**
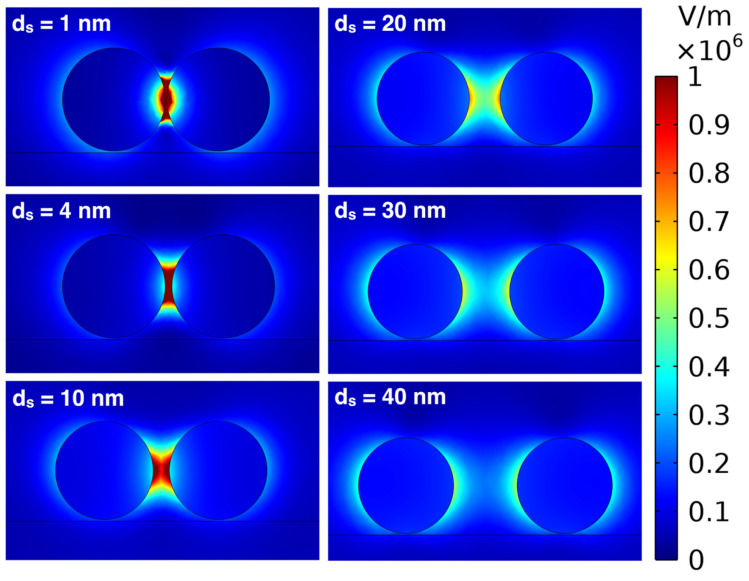
Images of the simulated distributions of electric field norm plotted for *d* = 60 nm AuNPs with *d_s_* = 1, 4, 10, 20, 30, and 40 nm.

In Figure 11 we can see that, in this case, when *d_s_* = 20 nm, the electric fields start to interact and at around *d_s_* = 10 nm, and the electric field is very high in the middle between the particles. The electric field further enhances as the distance between the *d* = 60 nm AuNPs decreases, and it is extremely high for very small distances (*d_s_* = 1 and 4 nm).

**Figure 12:**
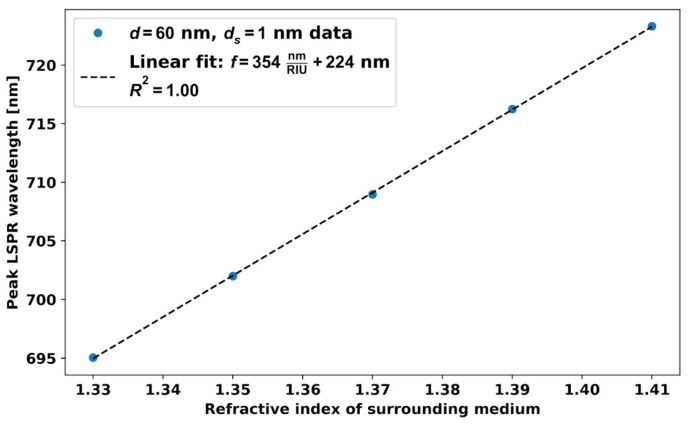
Resonance wavelength versus RI around the dimer of *d* = 60 nm AuNPs with *d_s_* = 1 nm gap.

Figure 12 shows resonance wavelength with RI around the 60 nm AuNP dimer separated by 1 nm. With this *d_s_* = 1 nm distance, the sensitivity is 2.8 times higher compared to the single *d* = 60 nm AuNPs, and it is 2 times higher compared to the AuNPs dimers of diameter *d* = 20 nm. The gap between the two nanoparticles (dimer) can be controlled by various methods, such as DNA origami [23,24] and solid phase approaches [25,26].

**Figure 13:**
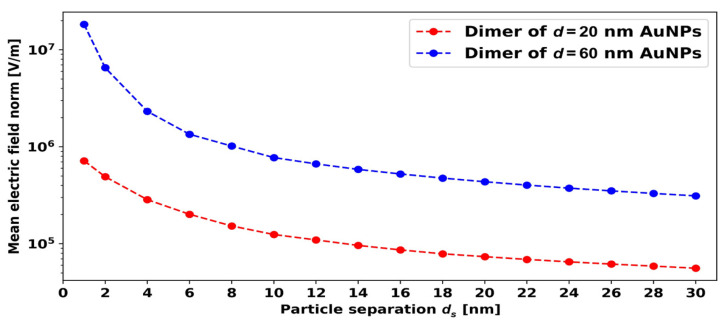
Mean electric field norm between AuNPs versus interparticle distance for the *d* = 20 nm and *d* = 60 nm AuNPs.

In Figure 13, the red and blue data points show the electric field variation in the middle between the particles as a function of *d_s_* for *d* = 20 nm and *d* = 60 nm AuNP dimers, respectively.

The electric field strength increases exponentially as the distance between the AuNPs in the dimer decreases. The *d* = 60 nm AuNPs provide the best FoM, and another advantage of using 60 nm AuNP dimers compared to 20 nm dimers is that maintaining the small distance (*d_s_* = 1–2 nm) between the AuNP dimer particles is not easy experimentally for small AuNP diameters. From an experimental point of view, the *d* = 60 nm dimer with a distance *d_s_* = 4–5 nm is more feasible compared to the *d* = 20 nm dimer with *d_s_* = 1–2 nm. Therefore, we can more practically use the *d* = 60 nm AuNP dimer with a distance *d_s_* = 4–5 nm, which also provides better sensitivity and FoM compared to *d* = 20 nm AuNP dimers with *d_s_* = 1 nm.

**Figure 14:**
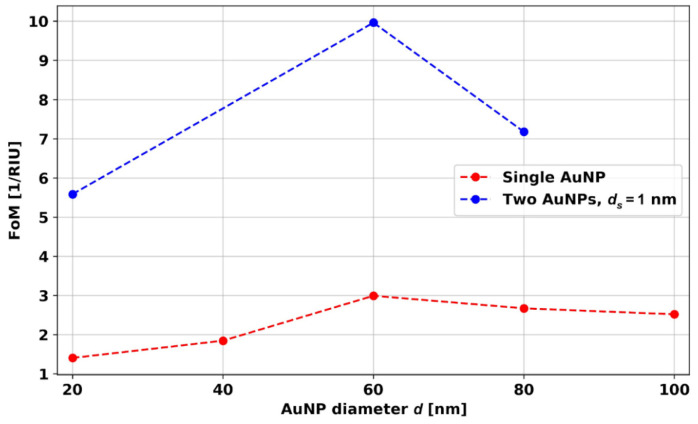
Simulated FoM for different size spherical AuNPs and AuNP dimers.

Figure 14 presents the simulated FoM for dimers of AuNPs with *d* = 20, 60, and 80 nm and *d_s_* = 1 nm. For the calculation of the FoM, the absorbance curve width was calculated at 80% of the maximum value of absorbance. The FoM of spherical AuNPs and dimers show a similar trend with *d*, i.e., the single AuNP with *d* = 60 nm and the dimer with *d* = 60 nm give the best FoM. The 3-fold enhancement in FoM can be achieved by maintaining the *d_s_* = 1 nm distance between the AuNP dimer particles.

A comparison of the sensitivity, FoM, and penetration depth of different sizes of AuNPs is presented in Table 1.

## 6. Conclusions

We studied several important performance parameters (sensitivity, FoM, penetration depth, and effect of interparticle distance) of spherical AuNPs in LSPR biosensing. The reported parameters are very useful for the advanced design of efficient LSPR-based biosensors. The RI sensitivity and FoM were investigated by simulations and experiments, while the penetration depth and effect of interparticle distance were simulated in COMSOL. Our observations show that 60 nm spherical AuNPs provide the best FoM. The interparticle distance plays a significant role in enhancing the sensitivity and electric field between the AuNPs. By controlling the distance between the AuNP dimer particles, for *d* = 20 and *d* = 60 nm, around a 3-fold enhancement in sensitivity and FoM can be achieved when compared to the performance of a single AuNP.

## Figures and Tables

**Table 1:** Comparison of different performance parameters of different sizes of gold nanospheres.

Diameter of AuNPs [nm]	Sensitivity [nm/RIU]	Figure of Merit (FoM) [RIU^−1^]	Penetration Depth [nm]	Ref.
15	44	0.6	Not mentioned	[13]
25	60	Not mentioned	Not mentioned	[15]
50	80			
100	180			
10–50	128–233	1.5–3.5	Not mentioned	[27]
60	126	Not mentioned	Not mentioned	[28]
60	97	1.62	18	[29]
20	51	1.4	5.47	Present work
40	68	1.8	11.65	
60	125	2.9	17.04	
80	140	2.6	21.84	
100	170	2.5	24.38	

## Data Availability

Data underlying the results presented in this paper are not publicly available at this time but may be obtained from the authors upon reasonable request.

## Supplementary Materials

The following supporting information can be downloaded at: https://www.mdpi.com/article/10.3390/mi14091717/s1, Figure S1. Picture of AuNPs in different solvents with average diameter (a) 20 nm, (b) 40 nm, (c) 60 nm, (d) 80 nm, and (e) 100 nm. Figure S2. Experimental absorbance spectra of AuNPs in different solvents with nanoparticles with average diameter (a) 20 nm, (b) 40 nm, (c) 60 nm, (d) 80 nm, and (e) 100 nm.
