# Supplementary material for: Investigation of Performance Parameters of Spherical Gold Nanoparticles in Localized Surface Plasmon Resonance Biosensing
Source: Micromachines (Basel). 2023 Aug 31;14(9):1717. doi: 10.3390/mi14091717 (PMC10535430; doi:10.3390/mi14091717)
Supplement: Supplementary file 1 [file micromachines-14-01717-s001.zip › micromachines-2548468-supplementary.pdf]

Supplementary file

Investigation of Performance Parameters of Spherical Gold Nanoparticles in Localized Surface Plasmon Resonance Biosensing

Vivek Semwal[1]*, Oliver Rishøj Jensen[2], Ole Bang[1] and Jakob Janting[1]*

[1]DTU Electro, Department of Electrical and Photonics Engineering, Technical University of Denmark, DK-2800 Kgs. Lyngby, Denmark
[2]Novo Nordisk Park, DK-2760 Måløv
*Correspondence: vivse@dtu.dk (V.S); jajant@dtu.dk (J.J)

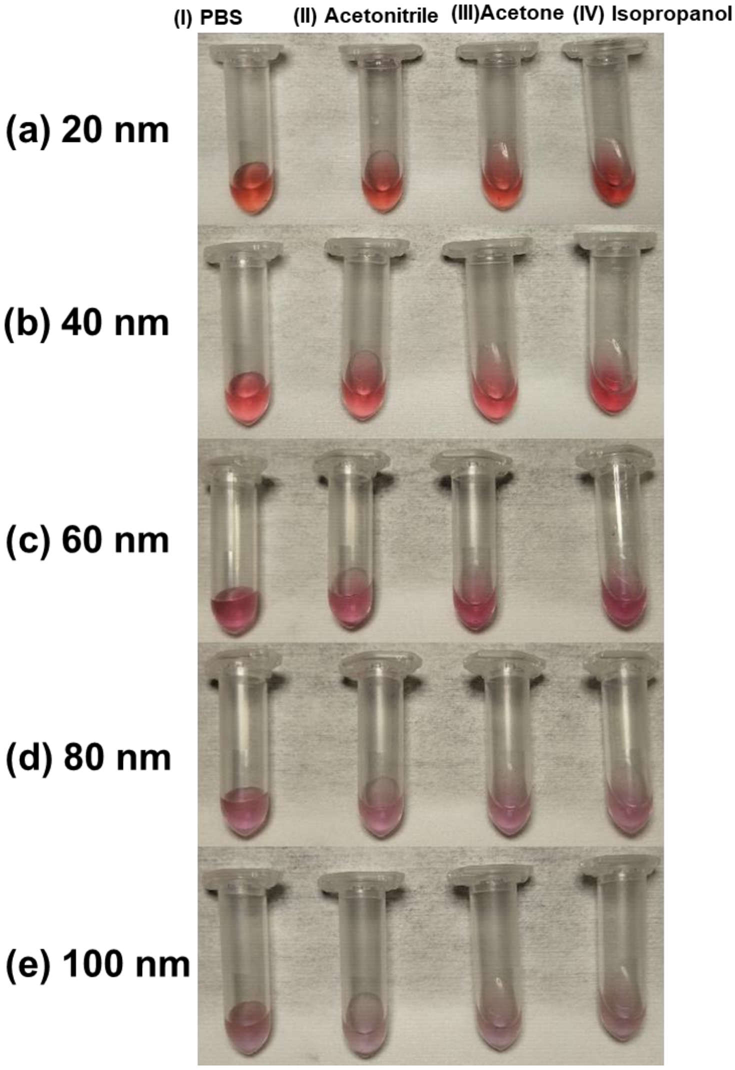

Figure S1. Picture of AuNPs in different solvents with average diameter (a) 20 nm, (b) 40 nm, (c) 60 nm, (d) 80 nm, and (e) 100 nm.

The absorbance curves of d = 20, 40, 60, 80 and 100 nm AuNPs at different RI mediums are shown in Figure S2.

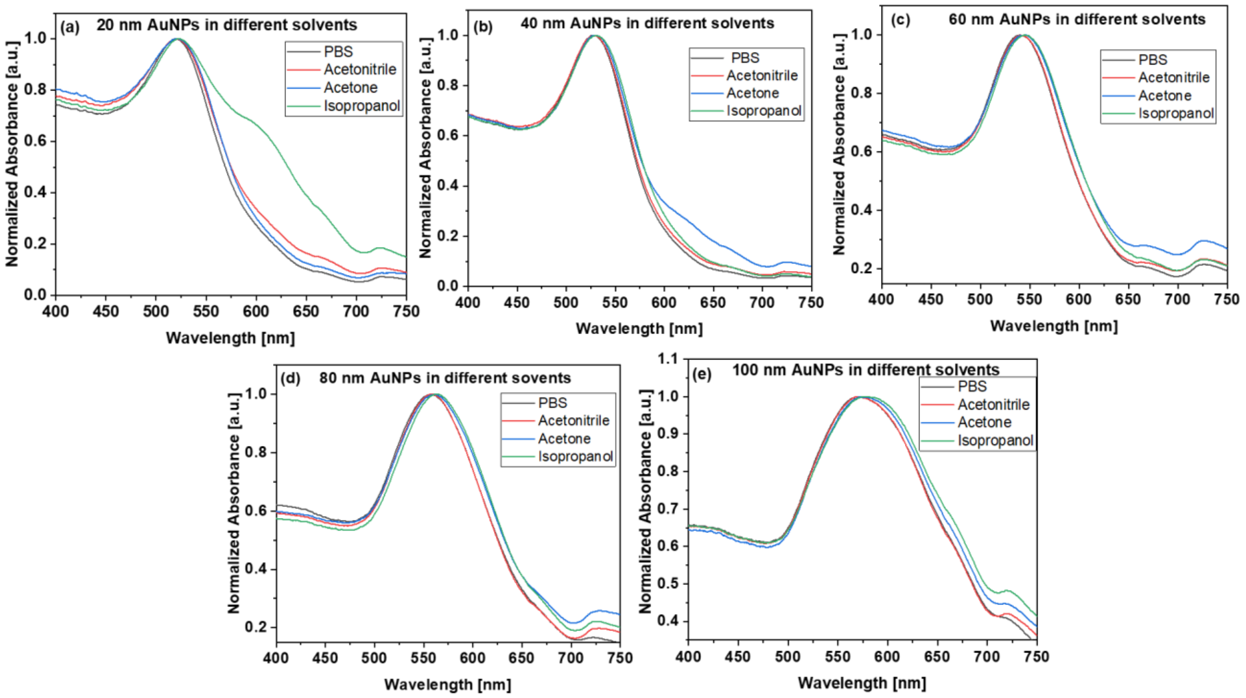

Figure S2. Experimental absorbance spectra of AuNPs in different solvents with nanoparticles with average diameter (a) 20 nm, (b) 40 nm, (c) 60 nm, (d) 80 nm, and (e) 100 nm.
